# The Welfare of Blinded Soldiers

**Published:** 1915-05-01

**Authors:** C. Arthur Pearson

**Affiliations:** Chairman, Blinded Soldiers' and Sailors' Care Committee. Blinded Soldiers' and Sailors' Hostel, St. Dunstan's, Regent's Park, N.W. (near Hanover Gate).


### THE EDITOR'S LETTER-BOX.

### The Welfare of Blinded Soldiers.

*To the Editor of* The Hospital.

Sir,—Judging from numerous letters which I have received lately a considerable amount of confusion has been created in the minds of those who are interested in the welfare of soldiers and sailors blinded in the war by the issue of circulars and advertisements from certain institutions for the blind which appeal for financial assistance to enable them to benefit these brave fellows.

These appeals have no connection with the establishment at St. Dunstan's, Regent's Park, which it has been my privilege to organise, and which is conducted with the concurrence and support of the War Office, the National Relief Fund, the Red Cross Society, the Order of St. John, and the National Institute for the Blind. Everything suggested by the most careful thought of men and women best qualified to advise as to the needs of the blind is to be found here, for the Blinded Soldiers' and Sailors' Care Committee has not depended upon its own unaided efforts, but has called in the assistance of experts in the treatment of the blind from all parts of the kingdom.

No ordinarily constituted institution for the blind is in a position to offer the blinded soldier or sailor the advantages which are open to him here. Everything which those responsible for the appeals in question propose to do in the more or less distant future, if the public will supply them with funds, is already being done at St. Dunstan's, including the provision of a seaside home situated at Brighton.

I am proud and happy to say that almost without exception those responsible for institutions for the blind are working in co-operation with us, and are helping in every way to arrange that blinded soldiers and sailors with whom they come in contact shall have an opportunity of participating in the unparalleled advantages which are open to them here. In my humble judgment this is not a matter into which competition should enter, nor does it seem decent to endeavour to induce the public to subscribe money for objects which it is perfectly well known are being adequately and successfully carried out already. —Yours faithfully,

C. Arthur Pearson,
Chairman, Blinded Soldiers' and
Sailors' Care Committee.

Blinded Soldiers' and Sailors' Hostel, St. Dunstan's, Regent's Park, N.W. (near Hanover Gate).
April 23, 1915.

[In this connection our readers will note with interest the report of the Ophthalmological Congress, on p. 107, in the course of which reference is made to certain cases which have been sent to St. Dunstan's Hostel.—Ed. The Hospital.]

